# Dynamic changes of paraoxonase 1 activity towards paroxon and phenyl acetate during coronary artery surgery

**DOI:** 10.1186/s12872-017-0528-z

**Published:** 2017-04-04

**Authors:** Anna Wysocka, Marek Cybulski, Henryk Berbeć, Andrzej Wysokiński, Janusz Stążka, Jadwiga Daniluk, Tomasz Zapolski

**Affiliations:** 1 Cardiology Department, Medical University of Lublin, ul. Jaczewskiego 8, 20–954 Lublin, Poland; 2 Internal Medicine in Nursing Department, Medical University of Lublin, ul. Jaczewskiego 8, 20–954 Lublin, Poland; 3 Biochemistry and Molecular Biology Department, Medical University of Lublin, ul. Chodźki 1, 20-093 Lublin, Poland; 4 Cardiosurgery Department, Medical University of Lublin, ul. Jaczewskiego 8, 20–954 Lublin, Poland

**Keywords:** Coronary artery bypass, Coronary heart disease, Paraoxonase 1

## Abstract

**Background:**

Serum paraoxonase 1 (PON1), an enzyme associated with high – density lipoproteins (HDL) particles, inhibits the oxidation of serum lipoproteins and cell membranes. PON1 activity is lower in patients with atherosclerosis and in inflammatory diseases. The systemic inflammatory response provoked during cardiopulmonary bypass grafting may contribute to the development of postoperative complications. The aim of the present study was to estimate the dynamic changes in paraoxonase 1 (PON1) activity towards paraoxon and phenyl acetate during and after coronary artery surgery.

**Methods:**

Twenty six patients with coronary heart disease undergoing coronary artery bypass grafting (CABG) were enrolled into the study. Venous blood samples were obtained preoperatively, after aortic clumping, after the end of operation, at 6, 18, 30 and 48 h after operation. Paraoxonase activity was measured spectrophotometrically in 50 mM glycine/NaOH buffer (pH 10.5) containing 1.0 mM paraoxon, and 1.0 mM CaCl_2_. Arylesterase activity was measured in 20 mM TrisCl buffer (pH 8.0) containing 1 mM phenyl acetate and 1 mM CaCl_2_.

**Results:**

PON1 activity toward paraoxon and phenyl acetate significantly decreased after aorta cross clumping and increased directly after operation. PON1 activity towards paraoxon in preoperative period and PON1 activity towards phenyl acetate in seventh stage of experiment tended to inversely correlate with the occurrence of postoperative complications.

**Conclusion:**

The paraoxonase 1 plasma activity is markedly reduced during CABG surgery.

## Background

Human serum paraoxonase 1 (PON1), a high density lipoprotein (HDL) associated serum esterase has been shown to be responsible for antioxidative properties of HDL. PON 1 is a 44 kDa, calcium dependent protein that remains associated with apolipoprotein apo-AI and apo-J on the particle of HDL. The enzyme is synthesized in the liver and secreted into the blood [1]. Recent studies have shown that HDL can prevent accumulation of lipid peroxides in LDL and PON1 enzyme is one of the compounds of HDL responsible for this activity. It has been reported that PON1 hydrolyzes the pro – inflammatory lipid peroxides generated by the oxidized low – density lipoprotein (LDL) reducing oxidative stress [2, 3].

PON1 activity is inversely related to atherosclerosis: it is lower in diseases accelerating development of atherosclerosis and it is reduced in inflammatory diseases [4, 5]. Deprivation HDL particles of PON1 as well as modification the biological function of enzyme due genetic recombination makes HDL unable to retard LDL oxidation, whereas returning PON1 to HDL restores its ability to inhibit LDL oxidation [6–8]. Several types of evidence suggest that low levels of PON1 protein raise the risk of development of premature atherosclerosis and low activity of PON1 is a strong independent risk factor for coronary heart disease [9–11]. A few studies demonstrate that serum PON1 activity decreases in patients with acute myocardial infarction [12, 13].

During cardiac surgery aorta cross-clumping and cardioplegic cardiac arrest induce global ischaemia, so cardiac surgery can be considered as human model of controlled ischeamia similar to this occurring in myocardial infarction. Moreover, cardiac surgery with cardiopulmonary bypass grafting provokes a systemic inflammatory response [14]. This inflammatory reaction may contribute to the development of postoperative complications including myocardial, renal or neurological dysfunction and respiratory failure.

The aim of the present study was to estimate the dynamic changes in PON1 activity towards paroxon and phenyl acetate during coronary artery surgery.

## Methods

### Study population

Twenty six unrelated individuals of Caucasian origin (men and women) aged 43–75 with coronary heart disease were included to this study. All patients had been admitted to Cardiosurgery Departament of Medical University in Lublin, for coronary artery by–pass grafting. The study protocol was approved by the local ethics committee (decision of Bioethics Committee of Medical University of Lublin No KE − 0254/76/2002). Written informed consent was obtained from all of the participants. The investigation conforms with the principles outlined in the Declaration of Helsinki.

The demographic data and a clinical history of risk factors were collected including age, gender, hypertension, smoking, diabetes mellitus, hypercholesterolemia and hypertriglicerydemia and family history of coronary heart disease. For each patient body mass index was calculated as body weight (kg) divided by the squared height (m^2^). There are also recorded ejection fraction (EF%) measured in echocardiography by the modified biplane Simpson method. Coronary heart disease was confirmed by positive coronary angiogram. Inclusion criteria were >50% narrowing of the lumen of at least one of the major coronary artery. The risk of operation was evaluated according to the Euroscore scale [15]. If the Euroscore was equal 1–2 points the risk of operation was regarded as low, if the Euroscore was equal 3–5 points as intermediate and in the case of score 6–13 points as high.

### Surgical technique and sample collection

All patients undergone coronary artery bypass with extracorporeal circulation. After standard general anesthesia a median sternotomy was carried out, followed by routine aortic and right atrial cannulation. Cardiopulmonary bypass was performed using heart – lung mashine SIII (Stockert) and moderate systemic hypothermia. Myocardial protection was achieved by mild hypothermic blood cardioplegia (32 °C). The degree of normovolemic hemodilution induced by a constant volume of priming (1800 mL) was determined on the basis of haematocrit measurements and body weight. During the procedure heparine was administered and the measured activated clotting time was >400 msek. After the end of the surgery heparin was neutralized with protamine and all patients were followed up in the intensive care unit. Data of aorta clumping and procedure time were collected. Venous blood samples were obtained before and after coronary bypass grafting at the following moments: 1) preoperatively, 2) after aortic clumping, 3) after the end of operation, 4) at 6 h, 5) at 18 h, 6) at 30 h, and 7) at 48 h after operation. Blood samples were anticoagulated with lithium heparin and separated from the cells by centrifugation.

### Biochemical analysis

Paraoxonase and arylesterase activities were determined according to Eckerson et al. [16]. PON1 activity towards paraoxon was determinated by measuring absorption at 412 nm with using continuously recording spectrophotometer (DU 640; Beckman) after introducing serum to 50 mM glycine/NaOH buffer (pH 10.5) containing 1.0 mM paraoxon, and 1.0 mM CaCl_2_. Enzyme activity was calculated with a molar extinction coefficient of 18,290 M^−1^ cm^−1^. One unit of paraoxonase activity produced 1 nmol of p- nitrophenol per minute. PON1 activity towards phenyl acetate was measured in 20 mM TrisCl buffer (pH 8.0) containing 1 mM substrate and 1 mM CaCl_2_. The absorbance was monitored spectrophotometrically at 270 nm. Enzyme activity was calculated with a molar extinction coefficient of 1310 M ^−1^ cm ^−1^. One unit of arylesterase acivity hydrolyzed 1 μmol of phenyl acetate per minute. The concentration of total plasma protein was assayed by biuret method. The relative PON1 activity towards paraoxon and phenyl acetate expressed in U per gram of protein was calculated.

Lipid profile was determined before surgery in blood samples collected through venipuncture in EDTA- coated tubes. Concentration of total cholesterol, triacylglycerols and HDL – cholesterol were tested by specific enzymatic techniques. LDL cholesterol was calculated from Friedwald formula.

### Statistical analysis

Data were statistically analyzed using the STATISTICA 9.0 (StatSoft Inc., Tulsa, OK, USA) for Windows software. The Wilcoxon paired test was used to compare mean values at each stage of experiment. The Mann-Whitney test was used to compare the differences of PON1 activity between groups according to the clinical features. Correlation was calculated using Spearman test. *P* values less than 0.05 were considered significant. Data on plots were presented as median (dot), 25%–75% percentiles (box), and minimum-maximum values (whiskers).

## Results

**Fig. 1:**
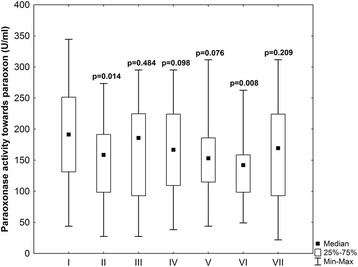
Paraoxonase activity towards paraoxon (U/ml) in subsequent stages of experiment (1) preoperatively, (2) after aortic clumping, (3) after the end of operation, 4) at 6 h, 5) at 18 h, 6) at 30 h, and 7) at 48 h after operation. *P*-values - preoperative data vs other stages of experiment (Wilcoxon test)

**Fig. 2:**
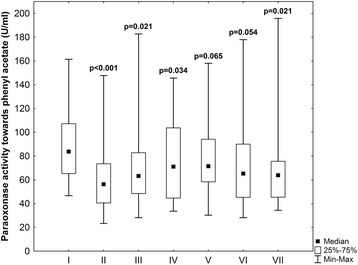
Paraoxonase activity towards phenyl acetate (U/ml) in subsequent stages of experiment: 1) preoperatively, 2) after aortic clumping, 3) after the end of operation, 4) at 6 h, 5) at 18 h, 6) at 30 h, and 7) at 48 h after operation. *P*-values - preoperative data vs other stages of experiment (Wilcoxon test)

Baseline and operative patients characteristic are reported in Tables 1 and 2. In the early postoperative period (to 48 h after surgery) serious postoperative complications were observed in 6 patients. There were two in-hospital deaths caused by perioperative myocardial infarct and cardiogenic shock and four patients developed left ventricle insufficiency. Paraoxonase activity towards paraoxon and phenyl acetate before, during and after CABG surgery is shown on Figs. 1 and 2. Statistical significance of changes observed in subsequent stages of experiment is reported in Table 3 (*p* values regard each pair of compared PON1 activity levels in previous and next stage of experiment). PON1 activity toward paraoxon in comparison with preoperative values decreased significantly (*p* = 0.014) after aorta cross clumping, increased directly after operation and then the tendency to decreased values in subsequent stages of experiment within 6 to 30 h after operation was observed to achieve the level similar to this one evaluated preoperatively at 48 h after operation. If compared with preoperative values significantly lower PON1 activity towards paraoxon was observed in the second (*p* = 0,014) and sixth (*p* = 0,008) stage of experiment. Comparison of PON1 activity between the next subsequent stages of experiment did not reveals significant differences between subsequent levels except from the tendency (*p* = 0.06) to higher PON1 activity after the end of the operation (third stage of experiment) in comparison with the time after aorta clumping (second stage of experiment). The tendency (*p* = 0.06) to increasing PON 1 activity towards paraoxon was observed between the sixth and the seventh stage of experiment. Similar differences were found after evaluating PON1 arylesterase activity. In comparison with preoperative period, PON1 activity towards phenyl acetate was significantly lower in all stages of experiment (*p* < 0,05) with exception of stages fifth and sixth, however in these stages the strong tendency to lower PON1 activities was found (p equal respectively 0.06 and 0.05). Also comparison of PON1 activity towards phenyl acetate between subsequent stages of experiment reveals the significant increase of enzyme activity in the third stage of experiment in comparison with the second stage (*p* = 0.01).

**Table 1:** Patients demographics and clinical data at baseline

Parameter	Value
Men (%)	80,77
Women (%)	19,23
Age (years ±SD)	61,2 ± 9,79
Hypercholesterolemia (%)	50,0
Hypertriglyceridemia (%)	26,92
Obesity (%)	23,08
Overweight (%)	42,31
Smokers (%)	34,62
Hypertensives (%)	57,69
Diabetes (%)	7,69
Family history of CAD (%)	7,69

*CAD* coronary heart disease

**Table 2:** Operative patients’ characteristics

Parameter	Value
Elective surgery (%)	69,23
Urgent surgery (%)	30,77
Ejection fraction (% ± SD)	53,1 ± 9,78
Patients with EF < 30% (%)	3,85
Patients with 30% ≤ EF <50% (%)	19,23
Operation risk (Euroscore) (±SD)	3,62 ± 2,43
Patients with low risk of operation (%)	26,92
Patients with intermediate risk of operation (%)	50,0
Patients with high risk of operation (%)	23,08
Number of grafts per patient (±SD)	3,5 ± 0,8
Number of arterial grafts per patient	1,0 ± 0,0
Number of venous grafts per patient	2,5 ± 0,7
Patient with implanted	
1 graft (%)	3,85
2 grafts (%)	26,92
3 grafts (%)	50,0
4 or more grafts (%)	19,23
Surgical time (min ± SD)	184,17 ± 51,54
Cross clamping time (min ± SD)	48,12 ± 13,31

**Table 3:** Comparison of PON 1 activity towards paraoxon and phenyl acetate, relative PON 1 activity in subsequent stages of experiment: (1) preoperatively, (2) after aortic clumping, (3) after the end of operation, 4) at 6 h, 5) at 18 h, 6) at 30 h, and 7) at 48 h after operation

Stage of experiment	Paraoxonase activity towards paraoxon	Relative paraoxonase activity towards paraoxon	Paraoxonase activity towards phenyl acetate	Relative paraoxonase activity towards phenyl acetate	Total plasma protein level (g/dL)
1	188.42 ± 79.28	3057.75 ± 269.58	91.56 ± 32.17	1483.74 ± 516.47	6.17 ± 0,78
2	146.39 ± 64.74 *p* = 0.01	2808.86 ± 1111.34 *p* = 0.32	59.63 ± 29.73 *p* < 0.001	1195.67 ± 663.62 *p* = 0.026	5.03 ± 0.70 *p* < 0.001
3	165.71 ± 77.10 *p* = 0.06	3105.23 ± 1370.27 *p* = 0.13	70.94 ± 36.24 *p* = 0.01	1339.40 ± 653.06 *p* = 0.04	5,28 ± 0.63 *p* = 0.02
4	166.82 ± 68.14 *p* = 0.67	3023.35 ± 1207.66 *p* = 0.46	75.26 ± 32.60 *p* = 0.69	1398.26 ± 693.96 *p* = 0.88	5.59 ± 0.76 *p* = 0.06
5	154.27 ± 66.50 *p* = 0.36	2779.60 ± 1259.10 *p* = 0.26	77.47 ± 29.27 *p* = 0.60	1374.70 ± 539.80 *p* = 0.98	5,69 ± 0.94 *p* = 0.67
6	138.44 ± 55.66 *p* = 0.42	2514.25 ± 1122.20 *p* = 0.3	75.30 ± 40.34 *p* = 0.13	1355.46 ± 708.89 *p* = 0.13	5.62 ± 0.88 *p* = 0.70
7	163.39 ± 82.29 *p* = 0.06	2901.89 ± 1567.51 *p* = 0.1	71.95 ± 40.48 *p* = 0.66	1351.55 ± 815.90 *p* = 0.66	5.65 ± 0.82 *p* = 0.34

Continuous data are reported as mean ± SD. Paraoxonase activities in subsequent stages were pair compared with Mann – Whitney U test. A *p* value in each stage presents comparison with the previous stage of experiment

**Fig. 3:**
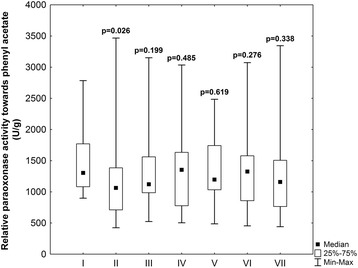
Relative paraoxonase activity towards phenyl acetate (U per g of total plasma protein) in subsequent stages of experiment: 1) preoperatively, 2) after aortic clumping, 3) after the end of operation, 4) at 6 h, 5) at 18 h, 6) at 30 h, and 7) at 48 h after operation. *P*-values - preoperative data vs other stages of experiment (Wilcoxon test)

Relative PON1 activity (per gram of total plasma protein) towards paraoxon did not differ significantly in subsequent stages (Table 3). Relative PON1 activity towards phenyl acetate was significantly decreased after aorta clumping (*p* = 0.026) in comparison with preoperative period (Fig. 3) and reached the same value as preoperatively in the third stage of experiment (*p* = 0.19) significantly increasing in the stage third in comparison with stage two (*p* = 0.04). The PON1 activity towards paraoxon throughout the study was not correlated with PON1 activity towards phenyl acetate (*p* > 0.05, Spearman test, data not shown).

**Fig. 4:**
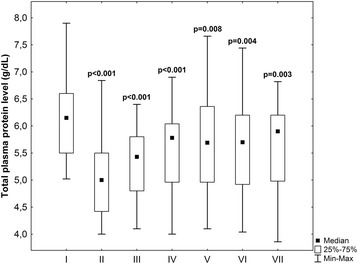
Total plasma protein level (g/dL) in subsequent stages of experiment: 1) preoperatively, 2) after aortic clumping, 3) after the end of operation, 4) at 6 h, 5) at 18 h, 6) at 30 h, and 7) at 48 h after operation. *P*-values - preoperative data vs other stages of experiment (Wilcoxon test)

Total protein plasma level significantly decreased (*p* < 0.001) after aorta cross – clumping and remained low (*p* < 0.01) in all stages of the experiment in comparison with preoperative value (Fig. 4). The protein plasma level after the end of operation raised significantly in comparison with the second stage and the tendency to higher values in fourth in comparison with third stage was observed. In other subsequent stages of experiment noted values of protein level did not differ significantly.

PON1 activity towards paraoxon in preoperative period (*R* = −0.341, *p* = 0.088) and PON1 activity towards phenyl acetate in seventh stage of experiment (*R* = −409, *p* = 0.056) tended to inversely correlate with the occurrence of postoperative complications. No correlation between PON1 activity in other stages of experiment and complications was found, although the correlation of protein level in the second stage with occurrence of complications (*R* = −0.402, *p* = 0.04) and similar trend in stage fifth and sixth (respectively *R* = −0.383, *p* = 0.05 and *R* = −0.344, *p* = 0.09) was notified (Table 4). Considering other established risk factors of unfavorable outcome after cardiac surgery, no correlation between PON1 preoperative value and ejection fraction, Euroscore scale, the time of operation and aorta cross clumping time was observed (*p* > 0.05, Spearman test, data not shown). Evaluating relationship of PON1 activity towards both substrates in next stages of experiment inverse correlation between PON1 activity towards phenyl acetate in the stage fourth and Euroscore scale result (*p* = 0.01 for absolute and *p* = 0.009 for relative activity) was found. The protein level after the end of operation correlate significantly with the time of operation (*p* = 0.01). Also the correlation between protein level and the time of aorta cross – clumping (*p* = 0.002 in the second stage, *p* = 0.04 in the sixth stage and *p* = 0.02 in the seventh stage) was noted.

**Table 4:** Correlation between paraoxonase1 activity and total plasma protein level with postoperative complications in patients after CABG in subsequent stages of experiment: (1) preoperatively, (2) after aortic clumping, (3) after the end of operation, 4) at 6 h, 5) at 18 h, 6) at 30 h, and 7) at 48 h after operation (R - correlation ratio and p - *p* value according to Spearman test)

Stage of experiment	Paraoxonase activity towards paraoxon	Relative paraoxonase activity towards paraoxon	Paraoxonase activity towards phenyl acetate	Relative paraoxonase activity towards phenyl acetate	Total plasma protein level (g/dL)
Stage 1					
R	-0,341	−0,28	-0,073	0,207	−0.219
p	0.088	0.166	0.723	0.31	0.282
Stage 2					
R	-0.313	−0.143	−0.01	0.09	−0.553
p	0.12	0.49	0.95	0.66	0.004
Stage 3					
R	-0.082	−0.164	0.111	0.181	−0.23
p	0.71	0.45	0.60	0.40	0.27
Stage 4					
R	-0.103	0.020	0.083	0.183	−0.401
p	0.61	0.91	0.67	0.37	0.04
Stage 5					
R	-0,317	−0.219	−0.257	−0.111	−0.383
p	0.11	0.28	0.216	0.60	0.05
Stage 6					
R	0,078	0.169	−0.097	0.026	−0.034
p	0.71	0.42	0.64	0.90	0.09
Stage 7					
R	0.049	0.091	−0.409	−0.037	−0.207
p	0.81	0.66	0.06	0.86	0.312

## Discussion

Our data indicate the significant decrease of PON1 activity after aorta cross clumping during CABG surgery. PON1 activity increased after surgery in comparison with values observed during operation, but the activity towards phenyl acetate remained lower than in preoperative period. Any previous studies evaluating dynamic changes in PON1 activity during cardiac surgery were not presented, yet. Findings achieved in this experiment may be connected with widely discussed processes of oxidative stress and inflammatory reaction associated with cardiac surgery and direct damage of myocardium during CABG. In previously presented data the unfavorable role of extracorporeal circulation (ECC) causing blood contact with non – physiological surface and using of cardioplegia during CABG are underlined, except from the surgical trauma itself.

PON1 hydrolyzes several different compounds including organic phosphates, carboxyl acids arylesters, aromatic carbonates, unsaturated fatty acids esters [17], but in laboratory practice, PON1 activity towards paraoxon (paraoxonase activity) and towards phenyl acetate (arylesterase activity) is usually assessed, as it was done in our study. PON1 activity is related to several genetic polymorphisms localized in coding and promotor region of PON1 gene. There were described two polymorphic forms of coding region of PON1 gene: substitution of glutamine (isoform Q or A) by arginine in position 192 (isoform R or B) and substitution of methionine (M) by leucine (L) in position 55. It was shown that PON1 isoenzymes hydrolyze some substrates in different rates depending on genotype. Hydrolytic activity towards phosphorganic compounds such as paraoxon is higher if the allel 192R is present. Alloenzymes QQ are more efficient in hydrolyzing neurotoxic substances as sarin or soman. Rate of hydrolysis of some substrates including phenyl acetate, naphtyl acetate or diazoxon is independent of genotype in position 192Q/R [18, 19]. In vitro studies have shown that PON1 QQ isoenzymes were more efficient hydrolyzing lipid peroxides in human atherosclerotic plaques derived from coronary and carotic arteries lesions [7]. A lot of studies investigating possible higher risk of CHD in individuals with 192R allel and protective role of 192Q allel were performed, but results were contradictory depending of evaluated population [20–23]. Another authors reported association between 55 M/L polymorphism and PON1 activity. It has been shown that in MM homozygous individuals the PON1 serum activity towards paraoxon is lower in comparison with LL homozygotes [24]. Additionally PON1 gene presents several polymorphism of promotor region: -108/C/T, −126G/C, −106A/G, −832 A/G and -909G/C [25]. It was proved that PON1 concentration and activity are affected almost exclusively by -108C/T polymorphism [26]. In our previous study [27] evaluating influence of PON1 activity and promotor region polymorphism (−108C/T) on short and long term outcome in 78 patients undergoing CABG we observed lower PON1 activity in the presence of TT genotype in comparison with CT and CC genotypes. Also other authors assessing larger group of patients reports that PON1 concentration and activity towards paraoxon were significantly lower in patients with CHD in comparison with healthy control group. In both investigated groups correlation of PON1 activity and -108C/T polymorphism was found with the highest enzyme efficiency in CC homozygotes and the lowest in TT homozygotes. In patients with CHD also relationship with PON1 concentration and −909 GC genotype was revealed [28]. Currently rather PON1 activity than polymorphism are regarded as a factor contributing to CHD occurrence. Thus reported in this paper differences in PON1 activity evaluating both paraoxonase as well as arylesterase activity can minimize the genotype influence on obtained results. Because the PON1 activity towards paraoxon is more affected by the occurrence of coronary heart disease and genetic polymorphism the observed changes in PON1 activity towards phenyl acetate may better express the pathophysiological circumstances of the cardiac surgery itself.

It was previously proved that tissue (also myocardium) ischaemia caused by ECC and blood flow decline are connected with increased level of oxidative stress, but the contribution of different components of oxidative and antioxidative balance (among them PON1) still remains unclear. Under circumstances of intensive oxidative stress plasma antioxidants level becomes lower. During the cardiac surgery using the ECC itself enhances the oxidative stress. Additionally at the time of reperfusion the tissue oxygene supply becomes rapidly restored and free oxygene radicals in amounts exceeding local antioxidative defense are formed. It was proved that in early postoperative period (hours after operation) in patients operated without using the extracorporeal circulation the level of hydroxylipids is significantly higher than in patients operated without using this device [29]. In another study serum concentrations of different oxidative compounds expressed as total antioxidant status, total oxidant status and oxidative stress index during on – pump coronary artery bypass grafting was assessed. Authors found significant increase of oxidative stress measurements after reperfusion. They concluded that oxidative imbalance may be associated with the aortic cross clumping time [30]. Another authors assessing changes in total antioxidant capacity of plasma in patients undergoing CABG found progressive depletion of evaluated values after the beginning of surgery, what remains in concordance with our results regarding PON1 activity towards phenyl acetate [31]. Even in the experiment evaluating influence or potentially less harmful phosphorylcholine – coated extracorporeal circulation system a higher oxidative stress with elevated antioxidant reaction were observed [32]. Also in our study we found a significant decrease of PON1 activity after aorta clumping and then an increase after the end of the operation when cardiopulmonary bypass was not still used, but we did not observed a correlation between PON 1 activity and time of aorta clumping. Our findings support the idea that using of ECC intensifying oxidative stress contributes to exhausting PON1 plasma supply. The reason of this phenomenon may be a result of PON1 gene expression inhibition and decreased protein synthesis in the liver. The decrease in PON1 activity may be concerned as analogy to another antioxidant enzymes depletion – superoxide dysmuthase (SOD) and glutatione peroxidase (GPx) – which activity decrease in enhanced oxidative stress was contributed to their inactivation by free oxygen radicals and products of their disintegration [33], although in the another study strong activation of SOD and GPx during ECC with the maximum level at the end of the cross clamp circulation was observed [34].

PON1 is a negative acute phase protein: PON1 plasma concentration rapidly decreases as a response on systemic inflammatory reaction. Acute phase activation during CABG is triggered by several agents such as surgical trauma itself, blood contact with extracorporeal surface, endotoxemia, tissue ischaemia. Blood contact with non physiological extracorporeal circulation surface leads to complement activation, release of pro – inflammatory cytokines (TNF–α, Il–6, Il-8, Il–13), leukocytes activation and adhesive molecules expression [14]. Pro – inflammatory cytokines unfavorably modify plasma lipid profile deteriorating protective function of HDL. Except from PON1 three other enzymes are responsible for antioxidative activity of HDL: lecithin cholesterol acyl transferase (LCAT), platelet activating factor acetylhydolase (PAF-AH) and above mentioned GPx, but the role of PON1 is considered as crucial. PON1 hydrolyzes peroxidation products contained in LDL particles, arterial walls and macrophages decreasing free radicals formation by NADH oxidase [35]. Modifications ongoing during acute phase reaction in HDL particle involve increase of free cholesterol content, incorporation of serum amyloid A (SAA) and ceruloplasmin and loss of apoJ, apoM and cholesterol esters [36, 37]. It was observed that in patients with septic shock and systemic inflammatory response syndromes apoM concentration significantly decrease and is reversely correlated to acute phase markers [38]. Additionally activity of several proteins attributable to HDL metabolism (LCAT, cholesterol esters transfer protein—CETP and hepatic lipase) and concentration of proteins responsible for antioxidative function of HDL decrease. Especially enzymatic activity of LCAT is considered as a factor involved in the protection of the formation of atherosclerotic plaques [39], but on the other hand it was shown that in vitro PON1 acts more efficiently than LCAT or PAF – AH protecting LDL against peroxidation [40]. Avian HDL, presenting PAF – AH activity, but devoid of PON1 do not protect human LDL against oxidative modifications [39]. As it was proved HDL particles modified as described above become pro-inflammatory and enhances oxidative stress [2]. It was shown that mentioned processes may occur during acute phase reaction, for example viral infection as well as chronic inflammatory process like atherosclerosis is regarded [41]. In several studies possible relationship between infective factors and acute coronary syndrome occurrence has been reported. It was noted that peridental infections or respiratory system infections increase the risk of myocardial infarction [42, 43]. Van Lenten et al. investigated abilities of HDL isolated from blood of patients 2–3 days after cardiac surgery in comparison with HDL obtained from healthy blood donors. HDL lipoproteins derivative from health persons inhibited LDL oxidative modifications and monocyte adhesion into endothelium cells. HDL from patients after cardiac surgery had pro – inflammatory abilities. In vitro loss of PON1 activity by HDL particles obtained from patients after CABG was proved [44]. During operations different from CABG PON1 activity decline was observed. Kumon et al. proved that PON1 activity decreases in patients after laparoscopic cholecystectomia. Blood samples were taken after 3,6 and 14 days after surgery. Decreased level of PON1 activity in comparison with preoperative period was observed in all stages of experiment despite of an increase of HDL and apo A-I concentration 14 days after operation. Authors suggest that PON1 activity decline maintaining so long may result from prolonging inhibition of PON1 gene expression and unfavorable modifications of HDL accompanied with acute phase reaction. Simultaneously modifying of PON1 activity by other acute phase proteins is also possible [45]. In the recent study it was reported that in a human experimental model of endotoxemia induced with endotoxin (LPS) intravenous administration subject with low HDL cholesterol were more susceptible to an inflammatory challenge. This response was independent of HDL cholesterol level in subject with the highest PON1 activity [46]. The latest phenomenon may contribute to slightly better prognosis in patients with higher PON1 activities, in which we observed postoperative complications less frequent.

Several factors is considered as predictors of worse outcome in patients undergoing CABG, most of them is included into Euroscore scale, but a clinical need to identify new factors that may allow for identification patients of higher risk of operation is underlined. From this reason we try to correlate investigated changes in PON1 activity with early postoperative complications. Poor outcome incidences observed in our study tends to be connected with the preoperative values of PON1 activity towards paraoxon and PON1 activity towards phenyl acetate in seventh stage of experiment, but no significant correlation with PON1 activity any other stage of experiment was found. In 4 patients we observed left ventricular insufficiency leading to need of circulation support or low cardiac output syndrome. In 2 patients myocardial infarction confirmed by increase of cardiac troponin >10 times above the normal level was recognized and these patients did not survive. The development of complications affecting cardiac function, as it was observed in our study, is commonly contributed to widely discussed above oxidative stress and inflammatory reaction. The balance between oxygen delivery and consumption in perioperative period is highly unsteady and any unsettling may lead to metabolic acidosis and deterioration of cardiac output [47]. For this reason preoperative low PON1 activity decreasing natural antioxidative defense may lead to imbalance resulting in impairment of cardiac function. As the main source of enhanced oxidative stress and inflammatory response during CABG seems to be ECC using, several studies evaluating influence of on and – off pump operation on outcome were performed and advantages of beating heart techniques was confirmed in terms of early mortality [48]. Providing of on – pump CABG was reported as a predictive factor of low cardiac output syndrome [49] and perioperative myocardial infarct [50], although the results of large randomized trials did not confirm the significant superiority off- pump technique [51]. Also in high operative risk patients treated preventively with intra – aortic balloon pump there was no significant difference in perioperative mortality in comparison with control group [52]. In our study the surgery was performed by conventional on – pump method and postoperative mortality (7.6%) and cardiac insufficiency (23%) did not depart from results of above quoted analyses (respectively 2–8,6% and 14–61% depending on operative risk of assessed population and severity of cardiac insufficiency). In recently published study investigating impact of inflammatory markers on clinical outcome, authors did not confirm differences between on – and off – pump group of patients regarding the occurrence of low cardiac output syndrome, postoperative myocardial infarction and death. Similarly like in our study, quoted authors reported correlation between assessed preoperatively levels of 8-isoprostaglandin F_2α_, asymmetric dimethyloarginine and β- thrombomoduline and postoperative complications [53]. Observed in our study correlation between total protein level and complications may be considered as being in accordance with the data indicating that in surgical operations including CABG decreased albumin concentration is related with less favorable outcome [54].

Cardiac surgery is reversibly connected with myocardium damage and even is considered as an experimental model of myocardial infarct. Observed in this study dynamic changes of PON1 activity may be compared to PON1 activity changes during myocardial infarct. Ayub et al. found that in patients with myocardial infarct after 2 h from beginning the chest pain occur a significant decrease of PON1 activity. Authors of quoted experiment do not reveal any significant differences of investigating parameters in postinfarctal period (1,2,3 days) [13]. Our data may support hypothesis that PON1 activity changes during myocardial infarct (or its experimental model: cardiac surgery) occur dynamically as a result of processes accompanied with myocardial ischaemia and should not be contributed only to previous decline of PON1 activity associated with atherosclerosis, because we did not find any significant correlation between PON1 activity and clinical (class of angina symptoms) or angiografic (occurrence of left main artery lesions) severity of coronary heart disease.

Discussing reasons of changes of PON1 activity during CABG process of hemodilution should be taken into consideration. Hemodilution is defined as an increase of liquid volume in the blood resulting in increase of plasma volume and decrease of blood red cells mass. During CABG a considerable amounts of liquids are given to supply loosen blood. This process may result in decrease of absolute PON1 activity directly after the heart function arrest and the retardation of the aorta blood flow. Storti et al. described that the level of total plasma protein decreases from 7,3 g/dl before to 4,8 g/dl after CABG. The level of total plasma protein remains decreased to 6 months after surgery [55]. In our study we also found decrease of total plasma protein concentration maintaining in postoperative period. Observed in our study differences of PON1 activity towards phenyl acetate were significant if expressed as PON1 activity per gram of plasma protein. That may indicate that process of hemodilution is not the only reason of changes of PON1 activity.

## Limitations

We are aware of several limitations regarding this study. The main is that investigated group of patients was relatively small - from this reason the frequency of noted complications were low, what undoubtedly influence the statistic analysis. We did not divide the group of patients with complications into subgroups according to cause and severity of cardiac insufficiency or used treatment (mechanical or pharmacological support, doses of inotropic drugs) like some other authors did, because statistical analysis of such small number of patients we consider as not valid. Future work should involve the larger group of patients and prolong the follow – up period.

### Conclusions

In conclusion our data indicate that PON1 activity is markedly reduced after CABG surgery. These findings support the hypothesis of the role involving oxidative stress and acute phase response in the myocardium damage during cardiac surgery. Informations collected in this study should encourage the development of strategies allowing to protect myocardium and prevent post – ischeamic damage.

## Abbreviations

CABG: coronary artery bypass grafting
EDTA: ethylenediaminetetraacetic acid
EF: Ejection fraction
HDL: High density lipoprotein
Il–13: Interleukin-13
Il–6: Intereukin-6
Il-8: Interleukin-8
LDL: Low density lipoprotein
LPS: Lipopolysaccharides
PON1: Serum paraoxonase 1
TNF–α: Tumor necrossi factor-α
